# Wild Argentinian Amaryllidaceae, a New Renewable Source of the Acetylcholinesterase Inhibitor Galanthamine and Other Alkaloids 

**DOI:** 10.3390/molecules171113473

**Published:** 2012-11-13

**Authors:** Javier E. Ortiz, Strahil Berkov, Natalia B. Pigni, Cristina Theoduloz, German Roitman, Alejandro Tapia, Jaume Bastida, Gabriela E. Feresin

**Affiliations:** 1 Instituto de Biotecnología-Instituto de Ciencias Básicas, Universidad Nacional de San Juan, Av. Libertador General San Martín 1109 (O), CP 5400, San Juan, Argentina; Email: jortiz@unsj.edu.ar (J.E.O.); atapia@unsj.edu.ar (A.T.); 2 Departament de Productes Naturals, Biologia Vegetal i Edafologia, Facultat de Farmàcia, Universitat de Barcelona, Avda. Joan XXIII s/n, 08028 Barcelona, Catalunya, Spain; Email: berkov_str@yahoo.com (S.B.); npigni@ub.edu (N.B.P.); jaumebastida@ub.edu (J.B.); 3 Facultad de Ciencias de la Salud, Universidad de Talca, Casilla 747, Talca, Chile; Email: ctheodul@utalca.cl; 4 Facultad de Agronomía, Universidad de Buenos Aires, Av. San Martín 4453, 1417, Buenos Aires, Argentina; Email: roitman@agro.uba.ar

**Keywords:** Argentinian Amaryllidaceae wild, alkaloids, galanthamine, lycorine, tazettine, acetylcholinesterase inhibitors

## Abstract

The Amaryllidaceae family is well known for its pharmacologically active alkaloids. An important approach to treat Alzheimer’s disease involves the inhibition of the enzyme acetylcholinesterase (AChE). Galanthamine, an Amaryllidaceae alkaloid, is an effective, selective, reversible, and competitive AChE inhibitor. This work was aimed at studying the alkaloid composition of four wild Argentinian Amarillydaceae species for the first time, as well as analyzing their inhibitory activity on acetylcholinesterase. Alkaloid content was characterized by means of GC-MS analysis. Chloroform basic extracts from *Habranthus jamesonii*, *Phycella herbertiana*, *Rhodophiala mendocina* and *Zephyranthes filifolia* collected in the Argentinian Andean region all contained galanthamine, and showed a strong AChE inhibitory activity (IC_50_ between 1.2 and 2 µg/mL). To our knowledge, no previous reports on alkaloid profiles and AChEIs activity of wild Argentinian Amarillydaceae species have been publisihed. The demand for renewable sources of industrial products like galanthamine and the need to protect plant biodiversity creates an opportunity for Argentinian farmers to produce such crops.

## 1. Introduction

Many species of medicinal and aromatic plants are cultivated for such industrial uses, but most are still collected in the wild. The demand for renewable sources of industrial products and the need to protect plant biodiversity create an opportunity for farmers to produce such plants as crops. More than 25% of the pharmaceutical drugs used in the World today are derived from plant natural products [1]. In the conventional pharmaceutical industry, pharmaceutical companies produce drugs from compounds extracted from plant material, or use plant derived compounds as starting material to produce drugs semi-synthetically [2]. Examples of the former include the anti-cancer alkaloid paclitaxel from Pacific yew (*Taxus brevifolia*), vinblastine from the Madagascar periwinkle (*Cataranthus roseus*), and digoxin from the foxglove (*Digitalis lanata*) [1].

The alkaloids of the Amaryllidaceae family are extensively studied for their biological activities in several pharmaceutical areas, for example, Alzheimer’s disease (AD), a neurodegenerative problem of enormous economic and social impact (15 million people, mainly in developed countries, suffer from the symptoms of this disease). The treatment is based on drugs that increase levels of acetylcholine. Galanthamine is a long-acting, selective, reversible and competitive inhibitor of acetylcholinesterase (AChE) and an allosteric modulator of the neuronal nicotinic receptor for acetylcholine. AChE is responsible for the degradation of acetylcholine at the neuromuscular junction, in peripheral and central cholinergic synapses. Galanthamine has the ability to cross the blood-brain barrier and to act within the central nervous system [3,4]. According to data presented by the Alzheimer’s Association in 2007, the prevalence of Alzheimer’s disease will quadruple by 2050. Galanthamine hydrobromide has superior pharmacological profiles and higher tolerance as compared to the original AChE inhibitors, physostigmine or tacrine [5]. This alkaloid galanthamine (biosynthesized exclusively by species of Amaryllidaceae family) is the treatment for mild and moderate stages of the AD. Galanthamine, approved in 2001 by FDA (Razadyne^®^), was originally isolated from *Galanthus woronowii*. While several total syntheses of the alkaloid galanthamine are available [6,7,8,9,10], current marketing is done mainly by the limited extraction of natural populations of *Leucojum aestivum* from Turkey (of varying quality and low content of active principle), or from small plantations of this species in Bulgaria, which are insufficient to meet current pharmaceutical company demand. The worldwide production of galanthamine is about 250 kg per year. Around 61 species of the Amaryllidaceae family grow in Argentina, covering a wide variety of genera (*Chlidanthus*, *Crinum*, *Habranthus*, *Haylockia*, *Hieronymiella*, *Hippeastrum*, *Phycella*, *Rhodophiala*, *Stenomesson* and *Zephyranthes*) [11]. To our knowledge, there are no reports on the chemistry and biological activity of Argentinian species belonging to the Amaryllidaceae group.

Our search for plant raw materials for medicinal products is now aimed at investigating the acetylcholinesterase inhibitory activity (AChE) of basic chloroform extracts (BCE) obtained from *Habranthus jamesonii*, *Phycella herbertiana*, *Rhodophiala mendocina*, and *Zephyranthes filifolia* (Amaryllidaceae species that grow in Argentine) to find new sources of production of galanthamine, and other potential alkaloids for treating AD. AChE inhibitory activity was determined by the spectrophotometric method by Ellman *et al.* [12]. Alkaloid profiles were analyzed by gas chromatography-mass spectrometry (GC-MS).

## 2. Results and Discussion

The AChE inhibitory activity of the BCE from *Habranthus jamesonii*, *Phycella herbertiana*, *Rodophiala mendocina* and *Zephyranthes filifolia* species, collected from the Andean region of San Juan (SJ), Mendoza (MDZ), and Neuquén (NQN) provinces (Argentine), were tested according to the methodology developed by Ellman *et al.* [12] with some modifications [13]. Galanthamine was used as a positive control. The results, expressed as IC_50_ values (µg/mL) are shown in Table 1*.* BCE showed the highest acetylcholinesterase inhibitory activity, with IC_50_ values ranging from 1 to 2 µg/mL (reference compound: galanthamine 0.29 ± 0.07 µg/mL). The BCE-*Z. filifolia* MZA and BCE-*H. jamesonii* SJ displayed the highest inhibition towards AChE with similar values (IC_50_ 1 ± 0.01 and 1 ± 0.08 µg/mL respectively) only three times higher than that of galanthamine. BCE-*P. herbertiana* SJ was found to have the second highest inhibition on AChE (IC_50_ values 1.2 ± 0.12 µg/mL). Acetylcholinesterase inhibition was similar to that of specie *R. mendocina* regardless of collection site (IC_50_ values 2 ± 0.15, 2 ± 0.20 µg/mL). BCE-*H. jamesonii* SJ showed a similar AChE inhibitory activity (IC_50_ values 2 ± 0.11 µg/mL). The yield percentages of the basic chloroform extract (BCE) (g/100 g dry bulbs) are reported in Table 1. BCE-*Zephyranthes filifolia* SJ had the lowest percentage at 0.21%, whereas BCE-*Rhodophiala mendocina* SJ gave the highest one at 0.38%.

**Table 1:** Acetylcholinesterase Enzyme Inhibition of Wild Argentinian Amaryllidaceae extracts expressed as IC_50_ [μg/mL].

Samples (voucher number)	BCE ^a^	
	Yield [%] ^b^	IC_50_ [μg/mL]
*Phycella herbertiana* SJ (IBT-Arg1)	0.34	1.2 ± 0.12
*Habranthus jamesonii* SJ (IBT-Arg2)	0.25	2.0 ± 0.11
*Rhodophiala mendocina* SJ (IBT-Arg3)	0.38	2.0 ± 0.15
*Zephyranthes filifolia* SJ (IBT-Arg4 )	0.21	1.0 ± 0.08
*Habranthus jamesonii* MZA (IBT-Arg5)	0.27	1.0 ± 0.01
*Rhodophiala mendocina* NQN (IBT-Arg6)	0.26	2.0 ± 0.20
Galanthamine ^c^		0.29 ± 0.07

^a^ Basic chloroform extract, ^b^ Percentage yield BCE [w/w], ^c^ Reference compound.

The alkaloids detected by GC-MS in the BCE-*H. jamesonii* MZA, BCE-*H. jamesonii* SJ, BCE-*P. herbertiana* SJ, BCE-*R. mendocina* NQN, BCE-*R. mendocina* SJ and BCE-*Z. filifolia* SJ are listed in Table 2.

**Table 2:** Alkaloid composition of four Amaryllidaceae plants.

Compound	*H. jamesonii*^ a^		*P. herbertiana*^ a^	*R. mendocina*^ a^		*Z. filifolia* ^ a^
	*SJ*	*MZA*	*SJ*	*SJ*	*NQN*	*SJ*
Trisphaeridine (**1**)	0.7			0.1		1.2
Ismine (**2**)						0.7
5,6-Dihydrobicolorine (**3**)						1.7
Galanthamine (**4**)	1.4	4.3	4.2	0.6	0.8	17.8
Lycoramine (**5**)	1.9		27.4	3.2		
Lycoraminone (**6**)			0.5			
Vittatine (**7**)	13.2		0.1	1.2	0.2	
Narwedine (**8**)		0.8	0.2		0.4	0.9
Anhydrolycorine (**9**)	2.5	1.5	0.4	0.9		
A-289 (**10**)	1.1					
A-315 (**11**)			0.4			
A-249 (**12**)	1.3	0.8				
A-319 (**13**)	1.1					
Montanine (**14**)	5.7		1.8	9.1		
Haemanthamine/Crinamine(**15**) ^b^	2.9		2.5	31.2	6.8	
Tazettine (**16**)	28.1		5.4	32.9		69.7
A-301 (**17**)	2.0					
Pancracine (**18**)				0.3		
11-Hydroxyvittatine (**19**)	18.7	3.1	4.6			
Galanthine (**20**)	4.9		17.2			
Lycorine (**21**)	8.2	43.6	33.2	13.3	20.4	
Incartine (**22**)			1.1			
Methylpseudolycorine (**23**)			0.2			
Epimacronine (**24**)				0.6		
8- *O*-Demethylhomolycorine (**25**)			0.6			
Homolycorine type (**26**)				3.9		
2- *O*-Acetyllycorine (**27**)			0.2	2.6		
A-345 (**28**)		2.7				
Tazettamide (**29**)		3.5				
*m/z* 109 (Homolycorine type) (**30**)		7.7				
Sanguinine (**31**)		0.5				
*m/z* 83 (285) (**32**)		1.1				
*m/z* 297(**33**)		11.8				
*m/z* 281 (**34**)		0.9				
*m/z* 283 (**35**)		2.3				
N-Demethylgalanthamine (**36**)					0.2	
2-O-Methylpancracine (**37**)					3.8	
N-Formylnorgalanthamine (**38**)					0.1	
Total Alkaloids identified	40.0	84.6	99.1	99.9	32.7	92.0

^a^ Values are expressed as GC-MS area %, ^b^ Cannot be distinguished by GC-MS.

**Figure 1:**
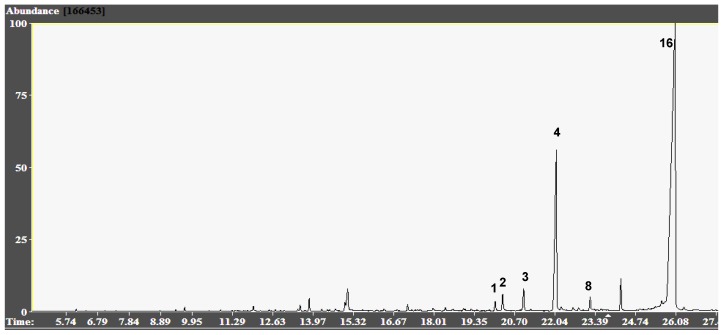
Representative GC-MS Chromatogram of Wild Argentinian Amaryllidaceae BCE-*Z. filifolia* SJ. Peaks: **1**: Trisphaeridine; **2**: Ismine; **3**: 5,6-Dihydrobicolorine; **4**: Galanthamine **8**: Narwedine; **16**: Tazettine.

Galanthamine (**4**) was found in all the species and it ranged from 0.6 to 17.8% of total ion current (TIC). *Zephyranthes filifolia* presented the highest galanthamine (**4**) content (17.8% TIC, Figure 1, whereas *R. mendocina* showed the lowest one (0.6% TIC). The highest AChE inhibitory activity of these species (IC_50_ 1.0 ± 0.08 µg/mL) belonged to BCE-*Z. filifolia* SJ a fact that could be related to the high content of galanthamine (**4**).

Galanthamine (**4**) content was collection site dependent: the BCE-*H. jamesonii* SJ sample presented 1.4% galanthamine TIC, while the BCE-*H. jamesonii* MZA with 4.3% TIC was four times higher. The differences in alkaloid content, depending on geographical distribution of *H. jamesonii* populations, coincides with a previous report on the European species. Berkov *et al.* [14], reported an intraspecies diversity in alkaloid profiles in *Galanthus elwesii* and *G. nivalis* populations collected in different locations in Bulgaria. They presented galanthamine TIC between 0 and 46%. The main alkaloid types (chemotypes) showed a wide variation in the number of compounds comprising their alkaloid mixture. Genetic and environmental factors and their interaction play a role in determining alkaloid profiles.

Additionally, sanguinine (**31**), identified in BCE-*H. jamesonii* MZA (0.5% TIC), has a hydroxyl group at C9 instead of a methoxyl group, and is around 10 times more active than galanthamine (**4**). Although the differential content could indicate that some environmental parameters might be influencing galanthamine (**4**) production, this specie could be considered for the sustainable production of galanthamine. A similar galanthamine (**4**) content (4.2% TIC) has been found in BCE-*P. herbertiana* SJ. BCE-*R. mendocina* SJ and BCE-*R. mendocina* NQN, showed a similar galanthamine content (<1% TIC). Narwedine (**8**), another AChE inhibitor [15] was found in all the populations studied (with the exception of the *H. jamesonii* and *R. mendocina* collected in San Juan province), but this compound comprised no more than 1% TIC. Other main alkaloids characterized by GC-MS in the BCE-*P. herbertiana* SJ were lycorine (**21**) (33%), lycoramine (**5**) (27%) and tazettine (**16**) (5.4%).

**Figure 2:**
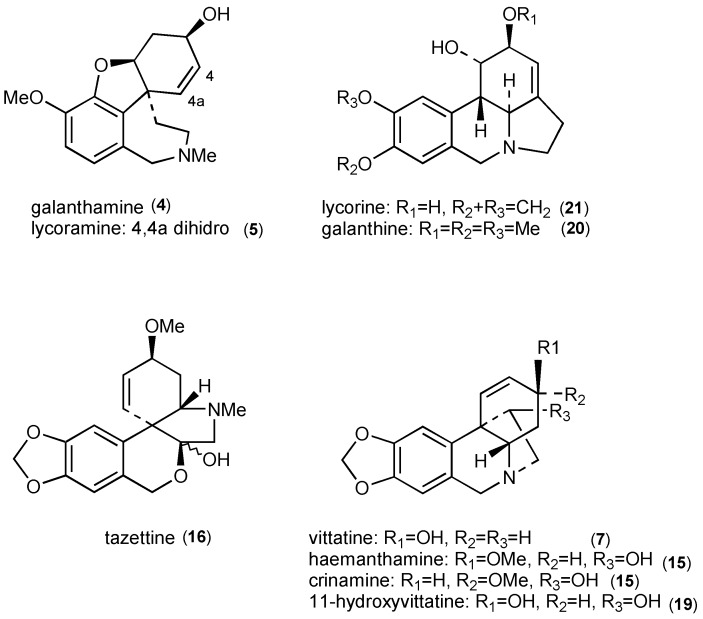
Main alkaloids in Wild Amaryllidaceae Species from Argentina.

The occurrence of trisphaeridine (**1**), galanthamine (**4**), lycoramine (**5**), vittatine (**7**), anhydrolycorine (**9**), montanine (**14**), haemanthamine/crinamine (**15**), tazettine (**16**), 11-hydroxyvittatine (**19**), galanthine (**20**), lycorine (**21**) and tazettamide (**29**) are reported for the first time in *Habranthus jamesonii* from Argentina. According to the literature, haemanthamine (**15**) and galanthine (**20**), have been reported previously in *Habranthus brachyandrus,* a specie of the genus [16]. At the same time, thirteen alkaloids were characterized in BCE-*R. mendocina* SJ, whereas five of them montanine (**14**), vittatine (**7**), haemanthamine (**15**), tazettine (**16**) and lycorine (**21**) have been previously reported as constituents of *Rhodophiala bifida* [17]. The main alkaloids identified in native Amaryllidaceae species from San Juan, Mendoza and Neuquén (Argentina) are shown in Figure 2.

**Figure 3:**
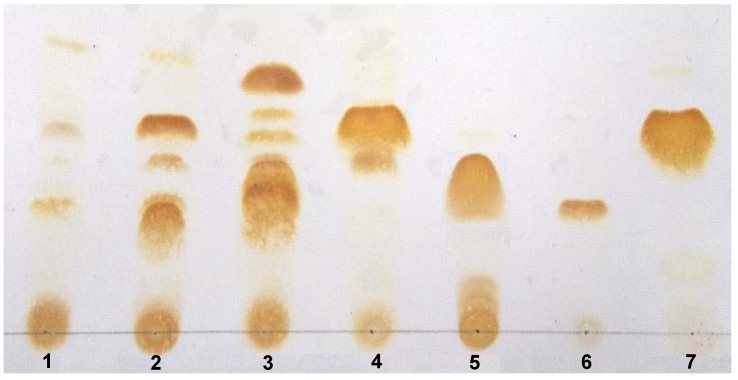
TLC Analysis of Argentinian Amaryllidaceae (BCE). **1**: *H. jamesonii*, **2**: *P. herbertiana*, **3**: *R. mendocina*, **4**: *Z. filifolia*, **5**: Galanthamine, **6**: Lycorine, **7**: Tazettine.

The presence of main alkaloids identified by GCMS (lycorine (**21**) and tazettine (**16**) as percentage of the total ion current (TIC) is consistent with that observed in TLC alkaloid profiles of chloroform basic extract (Figure 3).

The AChE inhibitory activity of these species can be explained by the presence of other AChE inhibitors in the alkaloid mixtures. Montanine (**4**) has shown significant AChE inhibitory activity [18], while a weak activity has been reported for lycorine (**21**) and haemanthamine (**15**) [19]. The other major alkaloids, lycoramine (**5**) and tazettine (**16**) have no AChE inhibitory activity [13] while, to the best of our knowledge, no AChE inhibitory activity assays have been performed for galanthine (**20**), 11-hydroxyvittatine, 2-*O*-acetyllycorine (**27**). 

The alkaloids found in the Argentinian species studied possess other interesting biological properties besides their AChE inhibitory activity. Haemanthamine (**15**) is a potent inducer of apoptosis [20], and has antimalarial activity [3]. Vittatine (**7**) has shown cytotoxic activity [3]. Antibacterial activity has been reported for vittatine (**7**) and 11-hydroxyvittatine (**19**) [21]. Lycorine (**21**) exhibits citotoxic, apoptotic, antiviral, antifungal, anti-protozoan [22], and anti-inflammatory activities [23]. It is a good candidate for a therapeutic agent against leukemia [24].Analgesic and hypotensive effects have been reported for galanthine (**20**) [3]. Moderate cytotoxic activity has been reported for tazettine (**16**) [25] which is an isolation artefact of chemically labile pretazettine. This compound, which is indeed present in plants, has shown remarkable cytotoxicity against a number of tumor cell lines [3]. In addition to the alkaloids identified in the species studied, other unknown compounds (**10**, **11**, **12**, **13**, **17**, **26**, **28**, **30**, **32**, **33**, **34**, and **35**) were detected in minor quantities, showing mass spectral patterns that also suggest structures related to the Amaryllidaceae alkaloids. Isolation studies are currently being developed.

## 3. Experimental

### 3.1. Plant Material

**Figure 4:**
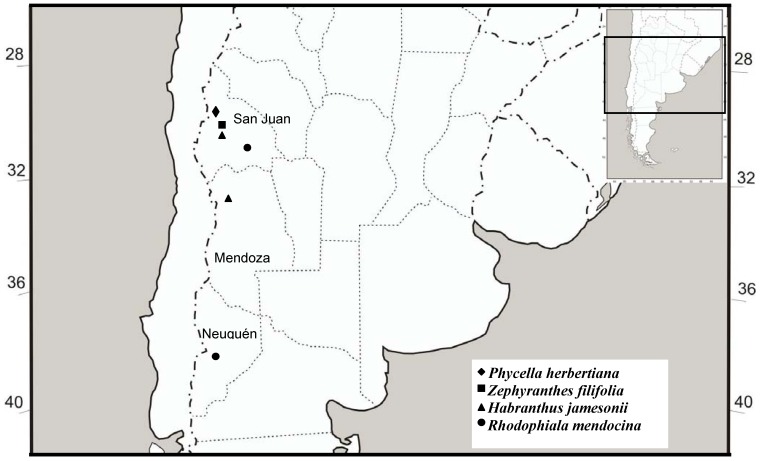
Collection areas of Argentinian wild Amaryllidaceae.

Wild plants of the species *Habranthus jamesonii* (BAK) Rav, *Phycella herbertiana* LINDL, *Rhodophiala mendocina* (PHIL.) Rav., and *Zephyranthes filifolia* (HERB.)ex Kraenzlin (Amaryllidaceae) were collected in the Andean regions of San Juan (SJ), Mendoza (MZA), and Neuquén (NQN) provinces (Argentina), during the flowering period between October and March 2009-2010 and then transferred to flowerpots and kept under greenhouse conditions. The species collected and identified, and voucher numbers are shown in Table 1. Figure 4 shows a map of the collection area. All plant species were authenticated by MCS German Roitman when they were collected. Voucher specimens were deposited at the Instituto de Biotecnología (UNSJ) with the codes: IBT-UNSJ- Arg1-6.

### 3.2. Alkaloid Extraction

Dried bulbs (100 g per each plant) were extracted under reflux three times with MeOH (300 mL) for 1 h each. The solvent was evaporated under reduced pressure to give the methanolic crude extracts (MCEs). MCEs were dissolved in H_2_SO_4_ (2% v/v) and neutral material was removed with CHCl_3_ (200 mL). Then, the aqueous solutions were basified with 25% NaOH up to pH 10-12 and the alkaloids were extracted with CHCl_3_ (3 ´ 500 mL) to obtain the basic chloroform extract (BCE). After evaporation of the organic solvent, the dry alkaloid fractions were dissolved in MeOH for GC/MS analysis. The BCE were named as BCE-*H. jamesonii* MZA, BCE-*H. jamesonii* SJ, BCE-*P. herbertiana* SJ, BCE-*R. mendocina* NQN, BCE-*R. mendocina* SJ and BCE-*Z. filifolia* SJ after their origin.

### 3.3. Gas Chromatography-Mass Spectroscopy Analyses

GC-MS analyses were performed on a Hewlett Packard 6890/MSD 5975 instrument (Hewlett Packard, Palo Alto, CA, USA) operating in EI mode at 70 eV. A DB-5 MS column (30 m ´ 0.25 mm ´ 0.25 μm) was used. The temperature program was: 100–180 °C at 15 °C min^−1^, 1 min hold at 180 °C, 180–300 °C at 5 °C min^−1^, and 1 min hold at 300 °C. Injector temperature was 280 °C. The flow rate of carrier gas (He) was 0.8 mL min^−1^. The split ratio was 1:20. The results obtained were analyzed using AMDIS 2.64 software (NIST). Compounds were identified through the comparison of their mass spectral patterns and retention indexes, with the data recorded in literature.

### 3.4. Microplate Assay for Acetylcholinesterase Activity

AChE activity was assayed as described by Ellman *et al.* [12] with some modifications [13]. Fifty μL of AChE in buffer phosphate (8 mM K_2_HPO_4_, 2.3 mM NaH_2_PO_4_, 0.15 M NaCl, 0.05% Tween 20, pH 7.6) and 50 μL of the sample dissolved in the same buffer were added to the wells. The plates were incubated for 30 minutes at room temperature before the addition of 100 μL of the substrate solution (0.1 M Na_2_HPO_4_, 0.5 M DTNB, 0.6 mM ATCI in Millipore water, pH 7.5). The absorbance was read in a Labsystems microplate reader (Helsinki, Finland) at 405 nm after three minutes. Enzyme activity was calculated as a percentage compared to an assay using a buffer without any inhibitor. The AChE inhibitory data were analyzed with the software package Prism (Graph Pad Inc., San Diego, CA, USA). IC_50_ values are means ± SD of three individual determinations each performed in triplicate.

### 3.5. TLC Analysis of BCE

TLC was carried out on Merck Silica gel 60 F254 plates, using chloroform-methanol-ammonia (99:9:1) mixtures as mobile phase. TLC plates were sprayed with Dragendorff’s reagent; main alkaloids gave orange spots.

## 4. Conclusions

The findings of the present study demonstrate the potential of wild Argentinian Amaryllidaceae species collected in the central Andean region, as a new renewable source of galanthamine. The most promising species seen to be *H. jamesonii* MDZ and *Z. filifolia* SJ. The demand for renewable sources of galanthamine and the need to protect plant biodiversity create an opportunity for Argentinian farmers to produce such crops. Studies of domestication of some of these species are currently in progress in order to determine which crops can be cultivated outdoors in the particular climate and soil, and which can be grown in greenhouses. Production cost and galanthamine levels in traditional cultivars are also being analyzed.
